# Oscillations in Absorption from InGaN/GaN Quantum Well to Continuum

**DOI:** 10.3390/nano15030174

**Published:** 2025-01-23

**Authors:** Marta Gładysiewicz-Kudrawiec, Mikołaj Żak, Witold Trzeciakowski

**Affiliations:** 1 Department of Experimental Physics, Faculty of Fundamental Problems of Technology, Wrocław University of Science and Technology, Wybrzeże Wyspiańskiego 27, 50-370 Wrocław, Poland; marta.gladysiewicz-kudrawiec@pwr.edu.pl; 2 Institute of High Pressure Physics, Polish Academy of Sciences, Sokołowska 29/37, 01-142 Warszawa, Poland; mzak@unipress.waw.pl

**Keywords:** InGaN/GaN p-i-n diode, infrared absorption, quantum well, electric field effect on intersubband absorption

## Abstract

We analyze theoretically an InGaN/GaN *n-i-p* diode with a single quantum well supporting only one bound state. The bottom parts of the diode, namely the first barrier and the quantum well, are heavily n-doped with silicon at 5 × 10^19^ cm^−3^ to ensure a high electron concentration in the well. The voltage drop in the diode occurs in the second AlGaN barrier, which is undoped, and structure ends with a p-doped GaN. The band structure of the diode is calculated by a Schrodinger–Poisson drift-diffusion solver. Next, we calculate the absorption from the bound state in the well to the “continuum” above the well. We show the oscillatory behavior of the spectrum, with the amplitude decreasing with more negative voltage applied to the diode. Oscillations are due to interferences of the wavefunctions between the edges of the well and the slope of the potential barrier.

## 1. Introduction

Intersubband transitions in quantum wells form the basis of Quantum Well Infrared Photodetectors (QWIPs) and Quantum Cascade Lasers, operating in the infrared [1]. These transitions occur for TM polarized light (perpendicular to the plane of the well so the samples require special preparation). For wider wells (supporting several bound states), the dominant transition is usually from the ground state to the first excited state. For narrow wells supporting only one bound state, the transitions to the continuum above the well become important. There is a rich literature describing the “above-barrier resonances” in various GaInAs/AlGaAs structures for wells close to the surface of the sample [2,3], double-barrier quantum wells [4,5] etc. These resonances occur due to interference of the continuum wavefunctions reflected from different potential discontinuities. Another class of “above barrier resonances” can be created by the presence of an electric field in the barrier. Electroabsorption in narrow GaAs quantum wells has been theoretically discussed in Refs. [6,7], and some experimental results were shown in Ref. [8]. The difficulties with experimental observation of the above-barrier electroabsorption resonances in arsenide structures were related to a fairly small effective mass of electrons (0.067*m*_0_ for GaAs), relatively weak fields (10–40 kV/cm) required for strong resonances, and wide barriers (over 50 nm) required between the wells. Since the rapid development of nitride devices, there have been many investigations of intersubband transitions, first in GaN/AlGaN quantum wells [9,10,11,12] and later in InGaN/GaN quantum wells [13,14,15]. The effective mass of electrons is much higher in nitrides (0.2*m*_0_ in GaN) and very strong electric fields can appear both in the wells and in the barriers (a few MV/cm). On the other hand, nitride quantum wells suffer from composition and width fluctuations which lead to much broader linewidths, both in the interband and in intersubband transitions. However, relatively narrow linewidths in intersubband absorption (38 meV) have been reported for GaN/AlGaN structures grown on nonpolar substrates [16].

In the present paper, we propose an *n-i-p* diode structure with a narrow InGaN quantum well (heavily doped) and with a strong electric field in the undoped barrier. This design provides much better conditions for observing absorption resonances than a quantum well in an external electric field. We calculate the potential profile of the structure under different external voltages using a one-dimensional drift-diffusion solver. The applied voltage allows us to tune the electric field in the undoped AlGaN barrier. Then, we calculate the absorption spectra from the ground state to the “continuum” above the quantum well. These spectra reveal the above-barrier resonances whose height and spacing changes with the applied voltage.

## 2. The Sample and Computational Methods

**Figure 1:**
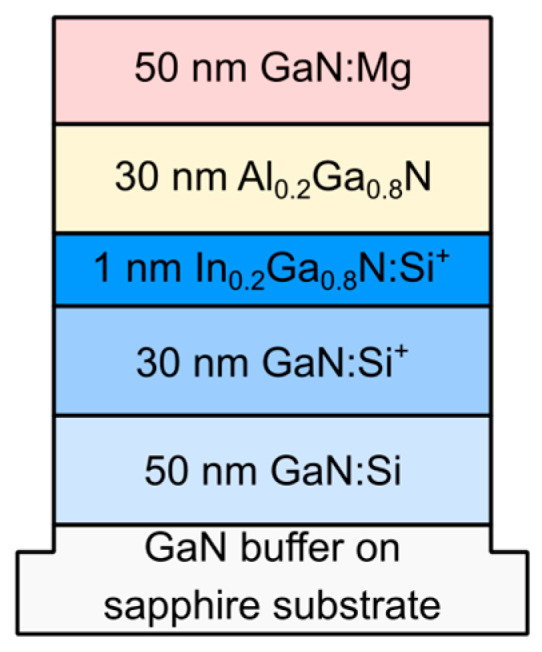
Composition and doping profile of our proposed diode.

The schematic structure of proposed diode is shown in Figure 1. The structure starts with a 50 nm wide GaN:Si layer doped at 5 × 10^18^ cm^−3^. Next, a 30 nm GaN barrier and 1 nm In_0.2_Ga_0.8_N quantum well are placed, both heavily n-type doped at 5 × 10^19^ cm^−3^. Subsequently, there is an undoped Al_0.2_Ga_0.8_N barrier (undoped, 30 nm wide) followed by 50 nm p-type GaN:Mg doped at 5 × 10^18^ cm^−3^. All the layers are assumed to be coherently strained and lattice-matched to GaN. The InGaN well is subject to about 1.8% in-plane compressive strain. The AlGaN barrier is subject to 0.5% in-plane tensile strain. The structure is set to be Ga-polar (0001), resulting in the formation of polarization charges at the interfaces. The diode can be grown on a sapphire substrate with a few micrometer thick GaN buffer layer, and then the p-type and n-type contacts are placed on top to enable the diode to be polarized by the external voltage. However, this voltage is kept below the turn-on voltage of the diode, thus we do not expect the device to heat up due to high current flow. Moreover, we do not expect significant heating of the diode during illumination by an infrared light, if placed on a proper heatsink.

The band structure and potential profile of the proposed *n-i-p* diode has been calculated with a one-dimensional Poisson–Schrodinger solver developed by Wu [17,18]. The Poisson equation which has been numerically solved (together with the Schrodinger equation) is as follows:

(1) $$\frac{\delta \varepsilon_{r}(z)}{\delta z}\frac{\delta V(z)}{\delta z}+\varepsilon_{r}(z)\frac{\delta^{2}V(z)}{\delta z^{2}}=-\frac{\rho (z)}{\varepsilon_{o}}$$

where *ε_r_(z)* is the relative dielectric permittivity, *V(z)* is the potential that is calculated as a result of successive iterations, and *ρ(z)* is the charge distribution in the structure. The charge distribution function is calculated from the electron density and the distribution of donors and acceptors in the structure. In simulations, we chose silicon (Si) as a donor and magnesium (Mg) as an acceptor for doping. The activation energies of those dopants are 25 meV for Si and 180 meV for Mg, respectively. The default material database has been used based on Refs. [19,20,21], and crucial material parameters can be found in Supplementary Materials.

**Figure 2:**
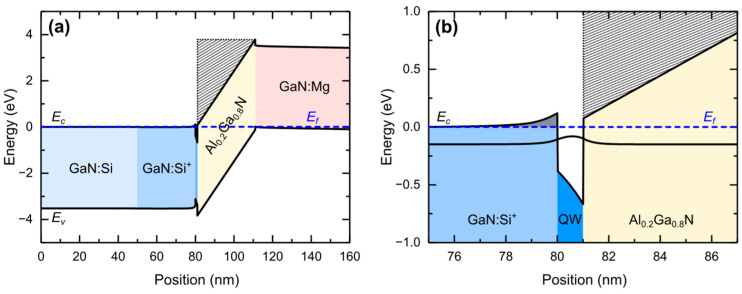
(**a**) Band structure profile of the diode at 0 V obtained from the simulation and (**b**) expanded region of the quantum well and barriers. The Fermi level *E_f_* is shown with dashed blue line. The hatched region above the right-hand barrier creates a triangular “well” which is the source of resonances in the “continuum”. The small barrier on the left-hand side (shown in grey color) perturbs the onset of “continuum”. The probability density of the ground state is shown in (**b**).

The band structure calculated for V = 0 V is shown in Figure 2a. The expanded region of the quantum well and barriers is shown in Figure 2b. Due to heavy doping in the left barrier and in the well, the built-in voltage of the *n-i-p* junction is mainly deposited in the undoped (right-hand) AlGaN barrier. The two-dimensional concentration of electrons in the well is determined as 8 × 10^12^ cm^−2^.

The idea leading to the choice of the above structure was to have a quantum well with only one bound state and the “continuum” for E > 0. In fact, the “continuum” in our structure is not infinite but bound in space by the buffer layer and the sapphire substrate. Therefore, we place an infinite barrier at z = 0 in Figure 2 so that the “continuum” extends between this barrier and the slope of the right-hand AlGaN barrier. The extension of the energy range of the “continuum” is limited by the height of the right-hand barrier in Figure 2, so it decreases with increasing positive bias. As it turns out, the results do not change if the infinite barrier is placed at z = 0, 10, 20, or 30 nm. Therefore, to speed up calculations, the infinite barrier was set at z = 30 nm. The additional barrier on the left-hand side of the well (colored in grey in Figure 2b in between 78 and 80 nm) perturbs the continuum but it is relatively small.

To calculate the absorption from the ground state to the continuum, we take into account the region between z = 30 and z = 110 nm. In this region, the Schrodinger equation (with the Duke Hamiltonian [22]) is solved numerically using the algorithm described in [23]:

(2) $$\left(\frac{\hbar^{2}}{2}\frac{\partial }{\partial z}\frac{1}{m\left(z\right)}\frac{\partial }{\partial z}+V\left(z\right)\right)\Psi \left(z\right)=E\Psi \left(z\right),$$

where *m*(*z*) is the effective mass along the z direction, and *V*(*z*) is the potential obtained from the one-dimensional Poisson–Schrodinger solver. After calculating the wavefunctions, it is possible to determine the absorption using the Fermi golden rule as follows:

(3) $$\alpha \left(\omega \right)=\frac{\alpha_{0}}{\omega }{\sum_{i,j}\left|M_{i,j}\right|}^{2}f\left(E_{i}\right)\left(1-f(E_{j})\right)\delta \left(E_{j}-E_{i}-\hbar \omega \right),$$

with $\alpha_{0}=\pi e^{2}/n_{r}\varepsilon_{0}m^{2}$, where *e* is the electron charge, $n_{r}$ is the refractive index, and $M_{i,j}$ is the matrix element corresponding to the transition between state *i* and state *j*. Similar calculation can be found in Refs. [24,25,26]. In the case of the intersubband transitions, the matrix element is nonzero for polarization along *z,* as follows:

(4) $$M_{i,j}=-i\hbar \int \Psi_{i}\frac{d}{dz}\Psi_{j}dz$$

In the considered case, the only occupied state is the ground state in the well, so the transitions are calculated from the state *i* = 1 to the excited states *j* > 1. In the plane of the quantum well, the states are plane waves (labeled by wavevector *k*) with parabolic dispersion, as shown in the following:

(5) $$E_{i}(k)=E_{i}+\frac{\hbar^{2}k^{2}}{2m(z)}$$

where *m* is the effective mass taken as 0.15*m*_0_ for the ground state (in the In_0.2_Ga_0.8_N well) and as 0.21*m*_0_ for the continuum states (which are mainly localized in the GaN barrier). This means that the transition energy depends on *k* and the summation over *k* states involves different one-dimensional states in the continuum.

The occupation of the ground state is governed by the Fermi function as follows:

(6) $$f\left(E\right)=\frac{1}{\exp\left(\frac{E-E_{f}}{k_{B}T}\right)+1}$$

where $E_{f}$ is the Fermi level, *k_B_* is the Boltzmann constant, and *T* is the temperature. We introduce level broadening [26,27,28] (caused by composition and width fluctuations in an InGaN well) by replacing delta function in Equation (3) with a Gaussian of a 40 meV half-width. We neglect the many-body effects [29,30,31,32], and we do not employ advanced DFT calculations [33,34]. Many-body effects due to Coulomb interactions (like excitonic effects) typically lead to corrections on the order of 10 meV, especially in the presence of a strong electric field in the well. In the presented scenario, electron–electron interactions can be omitted without significant impact. While the many-body effects may modify the absorption values, they do not alter the overall shape and the calculated position of absorption/gain curves [30]. Calculations excluding many-body effects remain in good agreement with the experimental results [35]. The many-body effects can induce excitonic phenomena [36] or other quasi-particle interactions such as polaritons [37] and plasmons [38], which significantly affect absorption spectra in two-dimensional materials or other non-classical semiconductors. However, in narrow InGaN quantum wells [29], these effects are small. As demonstrated in the next section, the absorption spectrum in our structure exhibits broad (100 meV) peaks due to the oscillatory behavior of the matrix elements. Under such conditions, the influence of electron–electron interactions (typically in the range of 10 meV) can be confidently disregarded.

## 3. Results

**Figure 3:**
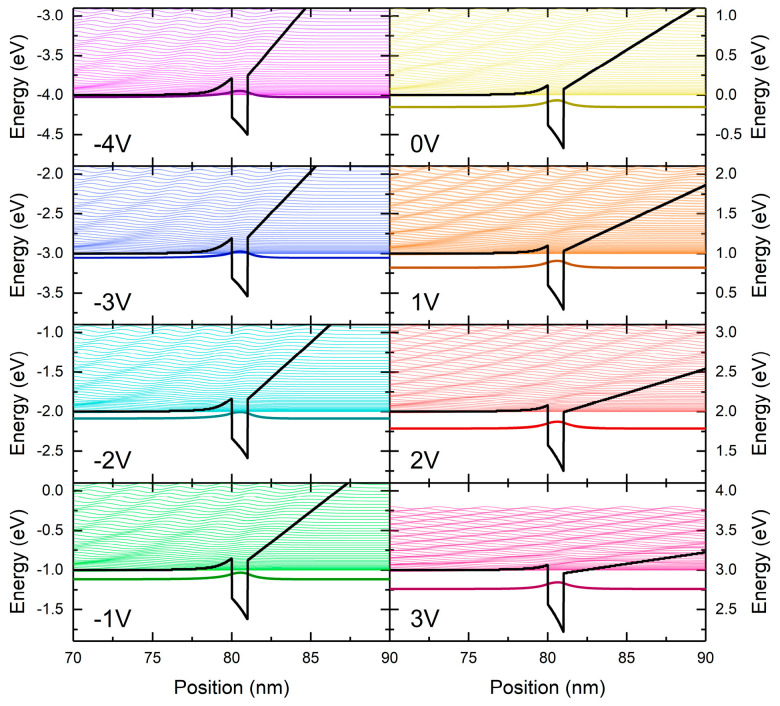
The conduction band profile and the wavefunctions for different voltages applied to the diode. At 3 V, the “continuum” range above the well is reduced.

The band structure profile shown in Figure 2 was used as input for calculating eigenstates, both within the well and in the continuum above it. An infinite barrier was added at z_0_ to obtain discrete states in the continuum, and we normalized them within this “box.” For the z_0_ values of 30 nm and below, the absorption spectra do not change significantly. Therefore, we chose z_0_ = 30 nm to achieve a reliable continuum simulation with not too many eigenstates (for numerical reasons). For z_0_ = 30 nm, the calculation of eigenstates and eigenfunctions was performed in the range within the continuum above the well determined by the AlGaN barrier. Some of the calculated wavefunctions are shown in Figure 3 for voltages from −4 V to 3 V applied to the diode. The field in the well increases while the field in the right barrier decreases with increasing voltage. Accordingly, the distance from the ground state to the onset of the continuum increases.

**Figure 4:**
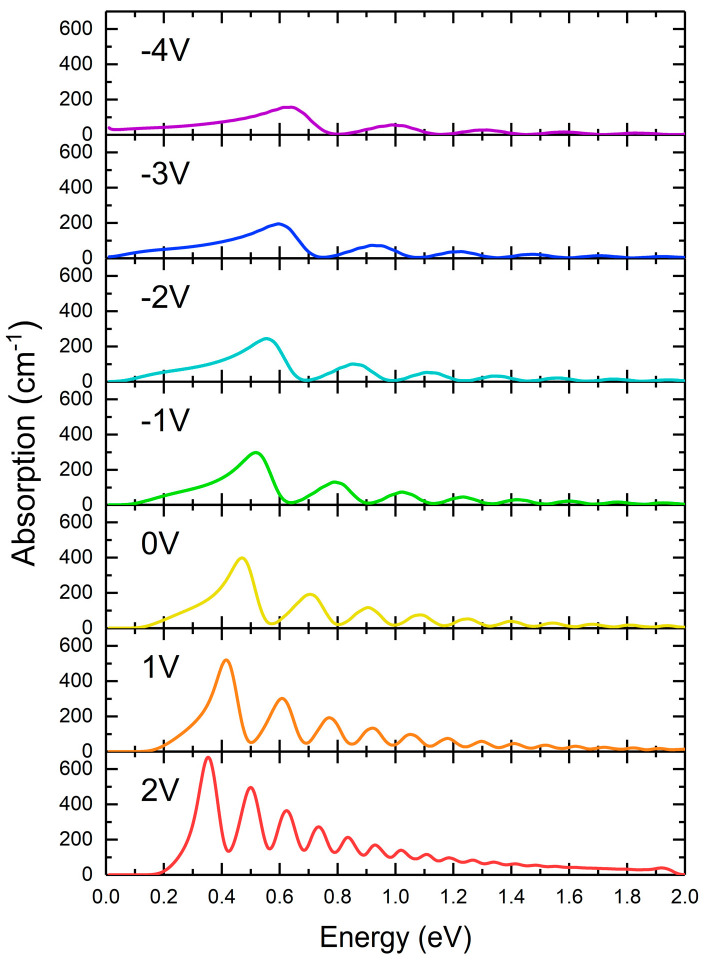
Absorption spectra of the diode for different applied voltages.

The absorption spectra for different voltages applied to the diode are shown in Figure 4. We observe a series of peaks, with decreasing separation and width with increasing voltage. The continuum range decreases for positive voltages since it is limited by the energy range corresponding to the right-hand barrier (see Figure 1). This is why we present the results up to V = 2 V since, as can be seen in Figure 3, the “continuum” range for V = 3 V becomes reduced (below 1 eV).

**Figure 5:**
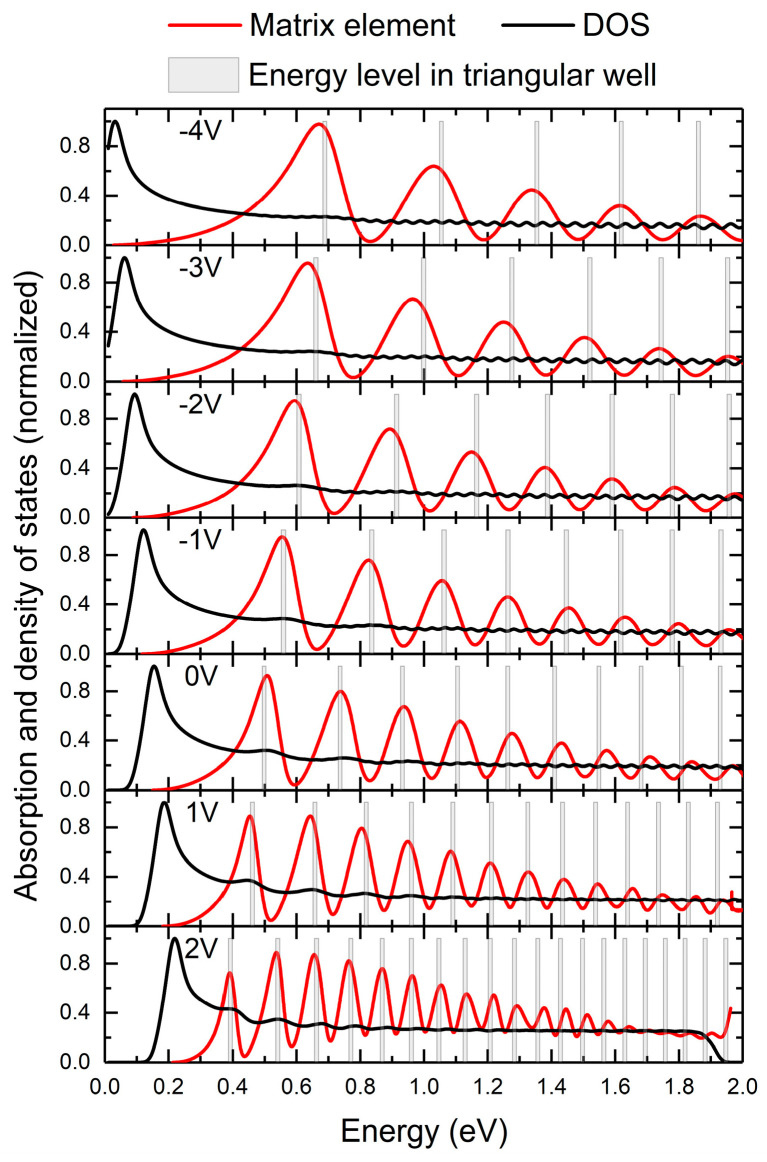
Momentum matrix element (red lines) and density of states (black lines) as a function of energy (calculated from the energy of the ground state) for different voltages applied to the diode. Bars show the position of bound states in a triangular “well” denoted in Figure 1.

In order to understand the origin of the spectra in Figure 4, we calculated separately the momentum matrix element (given in Equation (4)) and the density of the states measured from the ground state to the continuum. These are the two main factors affecting the absorption spectrum (Equation (3)). To facilitate comparison, the momentum matrix element and the density of states were normalized to dimensionless values by dividing them by their maximum value within the given range. These two quantities are presented as a function of the transition energy from the ground state to a given state in the “continuum”. The density of the states was calculated as the sum of the contributions from the individual transitions, which were defined as δ functions in Equation (3) and broadened to become Gaussians with a 40 meV half-width. The results are shown in Figure 5. The momentum matrix element (red lines) oscillates, and its maxima approach the continuum edge with increasing voltage. The maxima of the matrix element correspond approximately to bound states in the triangular well shown in Figure 2, i.e., the triangular quantum well with an infinite left-hand barrier and linear potential slope as the right-hand barrier (shown by bars in Figure 5). The downward shift of the red maxima with increasing voltage is simply due to the lowering of bound states in a triangular well with decreasing field. The density of states (black line) moves in the opposite direction with increasing bias, since the binding energy of the ground state increases with increasing bias (Figure 3). The absorption spectrum is dominated by the shift in the momentum matrix element, while the density of states produces the low-energy “tail” in the spectrum. Therefore, the resonances in the continuum are due to interferences of the wavefunctions reflected by the potential discontinuities at the edges of the quantum well and the reflection from the right-hand barrier. In order to increase the potential discontinuity at the right-hand edge of the well, we chose to have an AlGaN barrier in our diode.

**Figure 6:**
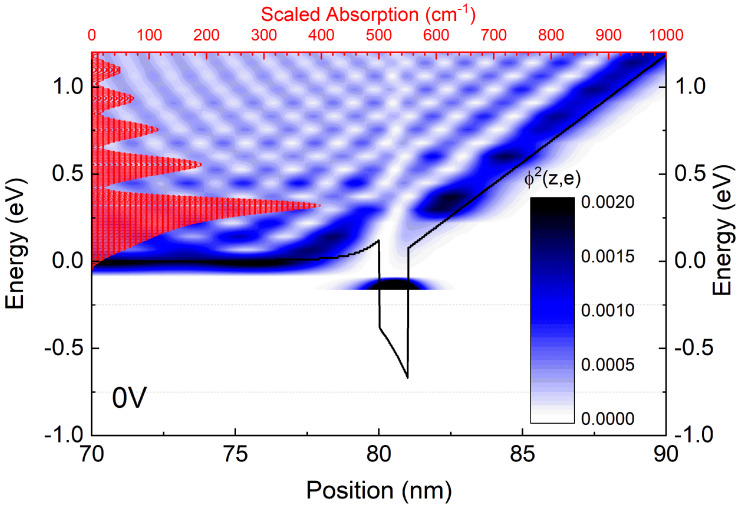
The probability density, shown as a color map depending on position and energy, calculated for V = 0. The black line represents the conduction band profile, while the red bar chart displays the rescaled absorption coefficient.

Figure 6 illustrates the probability density Φ^2^ plotted within the band profile for electrons in the case of V = 0. The probability is depicted using a color gradient from white through blue to black, depending on position and energy. The highest probability is observed within the quantum well. In the continuum, several regions within the triangular well exhibit increased probability. This result is compared with the previously calculated absorption, shown in red on the same figure as a bar chart. The absorption was rescaled (accounting for the difference from the ground state) to demonstrate that the transitions to the continuum correspond to the regions with the highest probability.

**Figure 7:**
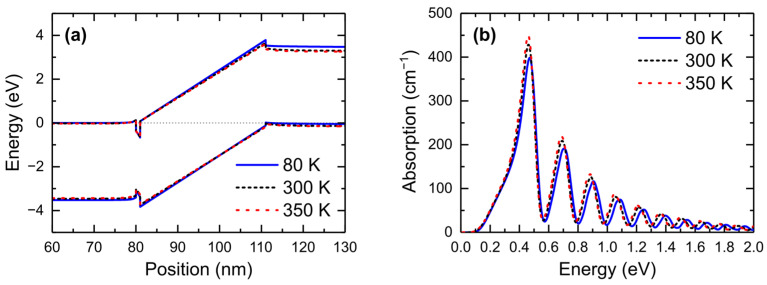
(**a**) Band structure and (**b**) absorption spectra of *n-i-p* diode at 0 V and 80 K, 300 K, and 350 K, respectively.

In the next step, we calculated the band structure and absorption spectra for V = 0 at the following three different temperatures: T = 80 K, T = 300 K, and T = 350 K (Figure 7). The three spectra turned out to be almost identical. This is because the dominant factor determining the broadening of the spectra is the momentum matrix element (given in Equation (4)) as can be seen by comparing Figure 4 and Figure 5. This implies that the possible heating of the sample by the light beam would not affect the oscillations in absorption.

**Figure 8:**
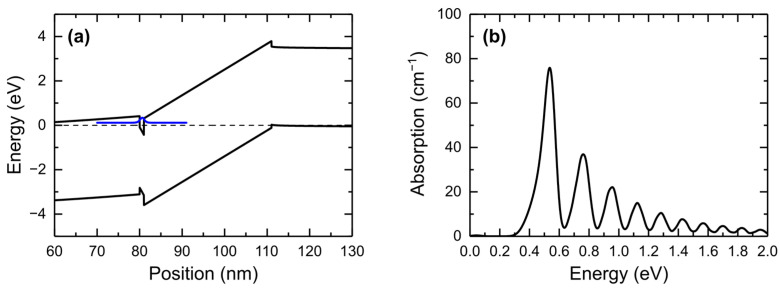
(**a**) Band structure and (**b**) absorption spectra of structure with Si doping at 10^17^ cm^−3^ in the quantum well and left-hand side barrier at 80 K and 0 V. The square of the bound state wave function in the quantum well is marked with a blue line.

Our proposed diode structure assumes very heavy doping in the left barrier and in the well. We checked the effect of much lower Si doping, namely 10^17^ cm^−3^ both in the left-hand barrier and in the quantum well. The potential profile and absorption spectra are shown in Figure 8. The left barrier becomes tilted and the concentration of electrons in the well is much lower than for the 10^19^ cm^−3^ doping. The oscillations are similar in terms of the period and width of the peaks, but the amplitude is about six times lower.

The additional calculation in the case of a nonpolar structure did not show absorption oscillations since the ground state in the well became resonant, so the electrons were not truly bound. Perhaps a deeper well (by increasing the indium content) or even more doping is needed to bind the ground state in a nonpolar quantum well.

**Figure 9:**
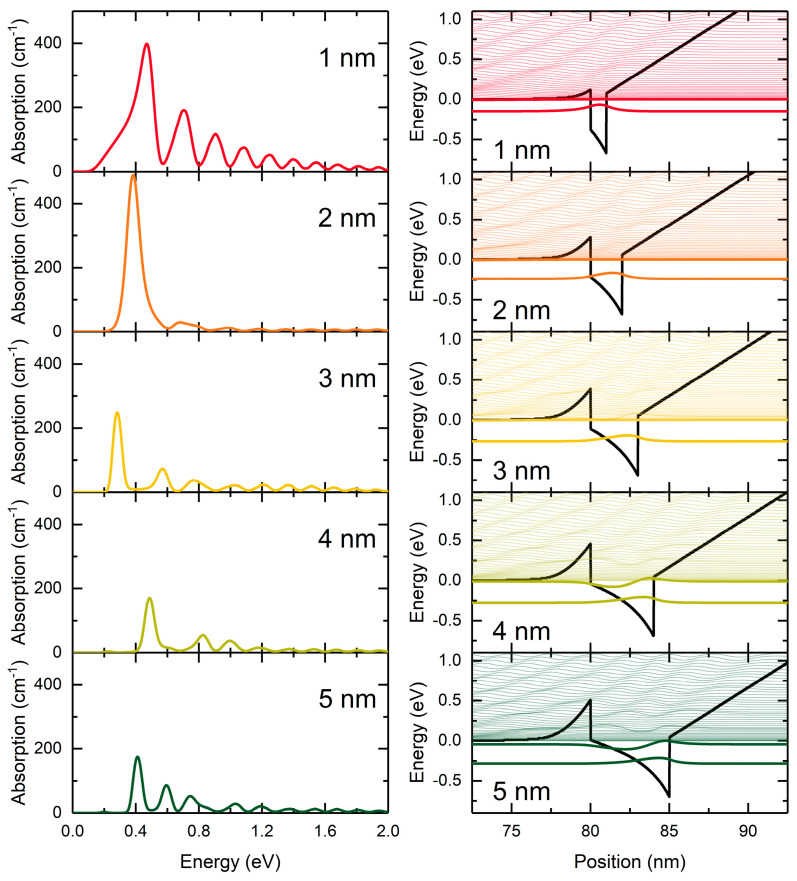
Absorption spectra for the diode from Figure 2 (at V = 0) for different widths of the In_0.2_Ga_0.8_N well and corresponding band profiles and wavefunctions.

We also checked how the absorption of our diode changed when we increased the well width in Figure 2. In Figure 9, we plot the absorption spectra (at V = 0) for single wells of increasing widths, together with the corresponding potential profiles. The highest absorption peak is observed for a well width of 2 nm, although the oscillations diminish rapidly. This behavior of the absorption coefficient stems from the calculated matrix element. The results indicate that the peak positions and widths primarily reflect the energy dependence of the matrix element. For wider wells, the intensity of the lines decreases. The left-hand barrier (colored with grey in Figure 2) increases with the increasing well width. Above the 4 nm well thickness, the second bound state appears in the well. This leads to the nonmonotonic dependence of the first peaks in the sequence (both in terms of intensity and position). Notably, the strongest absorption lines are observed for well widths of up to 3 nm.

## 4. Summary and Conclusions

We propose an InGaN/AlGaN diode structure which should enable the observation of oscillations in absorption from the ground state to the continuum. This is a better system for observing absorption oscillations than a single quantum well in an external electric field. Our calculation employs a semi-classical drift-diffusion/Poisson–Schrodinger solver for determining the band structure.

The case of InGaN/AlGaN quantum wells differs from the previously studied GaAs/AlGaAs structures due to higher effective mass and high built-in fields. However, the absorption from the ground state in a narrow well to the continuum above reveals similar oscillations as in GaAs wells. Resonances result from the interference of wavefunctions reflected from the edges of the well and from the right-hand side potential barrier. The absorption oscillations are the most pronounced for well widths below 3 nm, for which there is exactly one bound state. The amplitude and period of the oscillations can be tuned by an external voltage. The amplitude is greatest while the period is smallest for positive voltages of 2 V, that is, below the turn-on voltage of the *n-i-p* diode. Oscillations do not depend on temperature, as they mainly follow the maxima of the matrix element of momentum.

## Data Availability

Dataset available on request from the authors.

## Supplementary Materials

The following supporting information can be downloaded at: https://www.mdpi.com/article/10.3390/nano15030174/s1, Table S1: Material database used in one dimensional Poisson-Schrodinger solver developed by Wu [17,18] based on Refs. [19,20,21].
