# Supplementary material for: Oscillations in Absorption from InGaN/GaN Quantum Well to Continuum
Source: Nanomaterials (Basel). 2025 Jan 23;15(3):174. doi: 10.3390/nano15030174 (PMC11820425; doi:10.3390/nano15030174)
Supplement: Supplementary file 1 [file nanomaterials-15-00174-s001.zip › nanomaterials-3414880-supplementary.pdf]

Supplementary materials to

Oscillations in absorption from InGaN/GaN quantum well to the continuum

Marta Gładysiewicz-Kudrawiec [1], Mikołaj Żak [2], Witold Trzeciakowski [2,*]

Material database used in one dimensional Poisson-Schrodinger solver developed by Wu [17,18] based on Refs. [19-21].

**Parameter \ Material**	**GaN**	**$In_{0.2}Ga_{0.8}N$**	**$Al_{0.2}Ga_{0.8}N$**
Bandgap at 80K (eV)	3.5216	2.7256	3.9062
Bandgap at 300K (eV)	3.437	2.6472	3.8216
Bandgap at 350K (eV)	3.4092	2.6217	3.7932
m_e (m_0)	0.21	0.15	0.2255
m_{hh} (m_0)	1.87	1.8115	1.9903
m_{lh} (m_0)	0.14	0.1328	0.1542
P_{sp} (cm^{-2})	2.125e13	1.855e13	2.615e13
P_{pez} (cm^{-2})	0	-1.8635e13	4.4463e12
Band offset (-)	0.63	0.63	0.63
D (ε_0)	10.4	11.38	10.382

m_e – effective mass of electron; m_e – effective mass of heavy holes; m_e – effective mass of light holes; P_{sp} – spontaneous polarization; P_{pez} – piezoelectric polarization; Band offset defined as ratio of discontinuity of conduction to valence bands; D – dielectric constant
